# Phase II Randomized Study of Plitidepsin (Aplidin), Alone or in Association with L-carnitine, in Patients with Unresectable Advanced Renal Cell Carcinoma

**DOI:** 10.3390/md7010057

**Published:** 2009-03-05

**Authors:** Patrick Schöffski, Vincente Guillem, Margarita Garcia, Fernando Rivera, Josep Tabernero, Martin Cullell, Jose Antonio Lopez-Martin, Patricia Pollard, Herlinde Dumez, Xavier Garcia del Muro, Luis Paz-Ares

**Affiliations:** 1 University Hospital Leuven, Catholic University Leuven, Leuven, Belgium; 2 Instituto Valenciano de Oncologia, Valencia, Spain; 3 Instituto Catalan de Oncologia, Barcelona, Spain; 4 Hospital Universitario Marques de Valdecilla, Santander, Spain; 5 Hospital General Vall d’Hebron, Barcelona, Spain; 6 Pharma Mar S.A., Madrid, Spain; 7 NDDO Oncology, Amsterdam, The Netherlands; 8 Hospital Universitario 12 de Octubre, Madrid, Spain

**Keywords:** Aplidin, L-carnitine, plitidepsin, renal cell carcinoma, vascular endothelial growth factor

## Abstract

This randomized phase II study evaluated two schedules of the marine compound Plitidepsin with or without co-administration of L-carnitine in patients with renal cell carcinoma. Patients had adequate performance status and organ function. The primary endpoint was the rate of disease control (no progression) at 12 weeks (RECIST). Other endpoints included the response rate and time dependent efficacy measures. The trial also assessed the efficacy of L-carnitine to prevent Plitidepsin-related toxicity. The two regimes given as 24 hour infusion every two weeks showed hints of antitumoral activity. Disease control at 12 weeks was 15.8% in Arm A (5mg/m2, no L-carnitine) and 11,1% in Arm B (7mg/m2 with L-carnitine). Two partial responses were observed in Arm A (19 patients), none in Arm B (20 patients). Both schedules had the same progression-free interval (2.1 months). The median overall survival was 7.0 and 7.6 months. The safety profile was similar in both arms of the trial and adverse events were mainly mild to moderate (NCI CTC version 2.0). Increasing the dose to 7mg/m2 did not increase the treatment efficacy but the incidence of transaminase and CPK elevations and serious AEs. Coadministration of L-carnitine did not prevent muscular toxicity or CPK-elevation associated with Plitidepsin.

## 1. Introduction

Renal cell carcinoma (RCC) represents about 3% of all neoplasms. Most patients affected are adults between the ages of 50 and 70 years. The incidence of RCC is increasing at an annual rate of 2–4% [1, 2]. Around 80–85% are adenocarcinomas arising from the proximal tubules; the most common of these are clear-cell (75%) and papillary carcinomas (5–10%). Molecular studies have identified gene mutations in RCC, such as the von Hippel-Lindau gene (*vHL*) in clear-cell [3] and the *c-met* protooncogene in papillary carcinomas [4]. The function of the *vHL* is involved in the regulation of the vascular endothelial growth factor (VEGF), and alterations of the gene promote VEGF overexpression and hypervascularization of these tumors [5].

Surgery is the only available curative treatment and the stage of the tumor at initial diagnosis determines the prognosis [2, 6]. The 5-year survival of patients with advanced unresectable tumors is only 0–10%. Half of the patients will present advanced disease at some time and will be candidates for systemic therapy [7].

The traditionally used systemic treatments for RCC have been associated with poor effectiveness and appreciable toxicity. Hormone therapy has had minimal effects. Chemotherapy achieves response rates which do not justify its use (2, 8). Interferon-alpha (IFN) is associated with a response rate of 12% which increases to 30% in patients with predominantly pulmonary metastases and previous nephrectomy (8). High-dose intravenous (iv.) Interleukin-2 (IL-2) can induce 15–16% partial responses (PR) and 5 % complete responses (CR) [9] but is associated with significant toxicity. Combining i.v. IL-2 with subcutaneous IFN increases response rates but has no additional effects on survival [10].

The landscape for treatment of RCC is currently changing very rapidly. Newer options include the tyrosine kinase inhibitors sunitinib, sorafenib, the mTOR inhibitors temsirolimus and everolimus and the combination of bevacizumab with IFN. These treatment options, all of them with proven benefit in randomized Phase III trials, were not yet available when the current protocol was designed.

Plitidepsin (Aplidin) is a soluble marine product isolated from the tunicate *Aplidium albicans*. Aplidin induces apoptosis via activation of Jun N-terminal kinase, increases intracellular production of reactive oxygen species and alters the mitochondrial membrane potential. Plitidepsin reduces the transcription of the *ftl-1* gene, which encodes the VEGF receptor-1 in leukemia cell lines [11].

Plitidepsin has preclinical activity against a variety of tumor types (melanoma, non-small cell lung, prostate, ovarian and colorectal cancer) [12]. *In vivo*, plitidepsin showed efficacy against intraperitoneal melanoma and murine leukemia cell lines, and against human xenografts [13]. Several  phase I trials evaluated the safety and established recommended dose for different schedules, including a 24-hour infusion every two weeks and supplemented or not with L-carnitine. Phase I trials found signs of antitumor activity in colon, renal, head and neck, and lung carcinomas, melanoma, lymphoma and neuroendocrine tumors. When the present study protocol was written, 23 patients with RCC had already been treated with one of the regimens evaluated in phase I with clinical benefit in nine patients [14–17].

The most frequent dose-limiting toxicity (DLT) in phase I was muscular and characterized by reversible increases in serum creatine phosphokinase (CPK) levels frequently associated with cramps, myalgia and proximal weakness. Muscle biopsies found minimal or no necrosis, non-specific accumulation of glycogen and autophagocytic vacuoles and, in one patient, type II fiber atrophy. Gastrointestinal toxicity and liver toxicity were dose-limiting in other schedules. No hematological DLT’s were observed [14–17].

In one trial, four patients who had clinical benefit while receiving 24-hour iv. infusions every two weeks but developed muscular toxicity were treated empirically with L-carnitine. Plitidepsin inhibits palmitoyl thioesterase, which is related to carnitine palmitoyl transferases (CPT, mitochondrial enzymes that mediate the transfer of palmitate from the cytosol to the mitochondria). The myopathy induced by plitidepsin has clinical similarities with adult-onset CPT-2 deficiency. Administration of L-carnitine at an initial dose of 1.5 g three times a day, which is the dose recommended in the treatment of deficiencies in L-carnitine [21], allowed patients to continue receiving plitidepsin without any more disturbances. Prophylactic administration of L-carnitine increased the recommended dose for plitidepsin from 5 mg/m^2^ to 7 mg/m^2^. The most relevant toxicities were asthenia, vomiting, myalgia, constipation and diarrhea for the 5 mg/m^2^ dose, and asthenia, vomiting, diarrhea and transaminase elevation with co-medication L-carnitine [16].

The pharmacokinetic profile of plitidepsin administered as a 24-hour i.v. infusion every two weeks is characterized by dose linearity for area under the curve and maximum plasma concentration with doses up to 6 mg/m^2^, high interpatient variability and plasma clearance, long terminal half-life, and extensive distribution.

The promising profile found in phase I led to the conduct of this phase II study that assessed the efficacy and safety of plitidepsin in patients with advanced RCC. This study applied the two recommended doses established in phase I : 5 mg/m^2^ when given alone and 7 mg/m^2^ when combined with L-carnitine.

## 2. Results and Discussion

### 2.1. Patients and methods

#### Patient selection criteria

This randomized, open-label, multicentric phase II trial was approved by ethics committees in all involved institutions. It was performed between October 2001 and February 2006. Main eligibility criteria : age ≥18 years, ECOG performance status 0–1, life expectancy ≥3 months, histologically confirmed RCC in advanced or metastatic stage, documented disease progression in the past 6 months, measurable, not previously irradiated lesions and no more than 2 lines of previous systemic treatment. Biochemical selection criteria : neutrophils ≥1.5 x 10^9^/L, platelet count 100 x 10^9^/L, creatinine ≤1.5 upper limit of normal (ULN) or a clearance of ≥40 ml/min, serum bilirubin ≤ 1.5 mg/dL, alkaline phosphatase ≤2.5ULN (up to 5 x ULN in case of bone metastases), transaminase <2.5ULN, and albumine >25 g/L. Patients were excluded if they had transitional cell carcinoma, nephroblastoma or sarcomatoid tumors, other previous malignancies, brain metastasis, hypercalcemia, myopathy or CPK elevation endocrine diseases or fructose intolerance.

#### Study design and endpoints

A total of 38 patients were randomized to receive plitidepsin either alone (Arm A) or combined with oral L-carnitine at a dose (Arm B). The sample size was based on a one stage Fleming design with a type I error of 0.1, a power of 90%, a PFS rate at 12 weeks for the null hypothesis of disease control rate=20% and for the alternative hypothesis =50. Treatment lasted until disease progression, unacceptable toxicity, prolonged treatment delay, protocol violations or patient’s refusal. The primary endpoint was the objective rate of disease control, defined as the percentage of patients who were free of disease progression at 12 weeks. This endpoint is infrequently used in RCC trials but very common in other settings such as sarcoma. Secondary endpoints included the rate of objective responses and time-related endpoints. Response was evaluated using the Response Evaluation Criteria In Solid Tumors (RECIST) at eight weeks after treatment onset in patients who had received at least two infusions; absence of progression had to be confirmed with additional evaluations conducted four weeks later (*i.e.*, at week 12) and then once every 8 weeks.  Adverse events were evaluated using the National Cancer Institute Common Toxicity Criteria (NCI-CTC, version 2.0).

#### Drug administration and concomitant medication

Plitidepsin was administered as a 24-hour i.v. infusion every two weeks using a central or peripheral iv. line, either at a dose of 5 mg/m^2^ or at a dose of 7 mg/m^2^, the latter combined with 1.5 g of oral L-carnitine given every 8 hours. Each administration of plitidepsin was considered a cycle. Assignment to the treatment arms was randomized. L-carnitine started on the first day of treatment and continued up to 14 days after the last plitidepsin infusion. The plitidepsin dose could be reduced in the event of clinically relevant hematological and non-hematological toxicities. Patients were eligible for retreatment if they had recovered from all hematological toxicities, liver and muscular toxicity. If no recovery occurred, treatment was delayed for up to two weeks. Afterwards patients had to be withdrawn.

Plitidepsin could be reduced in Arm A from 5 mg/m^2^ to 4 and 3.2 mg/m^2^ respectively, in Arm B, to 6 and 5 mg/m^2^. The dose of L-carnitine given to the patients in Arm B could be tapered off in the event of digestive discomfort. The protocol did foresee the following dose levels for L-carnitine : 4.5 g/day, 4.0 g/day and 3.0 g/day divided in 3 daily portions.

All concomitant medications were recorded. Patients received prophylactic anti-emetics (dexamethasone, setrone, metoclopramide). Patients in Arm A who developed grade 2 or greater muscular toxicity were allowed to receive treatment with L-carnitine until it decreased to grade 1 or less. Secondary prophylaxis with L-carnitine and continuing the treatment with plitidepsin without dose reduction was not allowed.

#### Diagnostic process

The following study procedures were conducted before the start of treatment: history, clinical examination, hematology and biochemistry, urinnalysis, chest X-ray and/or CT/MRI scans of all sites with measurable/evaluable lesions, and ECG. Objective progression at baseline had to be documented.

On study procedures : clinical examination, hematology and biochemistry, toxicity/symptoms, tumor imaging. Patients were followed for toxicity until the resolution of all AEs. All patients who received at least one infusion and were evaluated at eight weeks were to be considered evaluable for efficacy. Patients who received at least one infusion were evaluable for safety

The absence of progression had to be confirmed by imaging conducted at week 12. The duration of response was calculated from the date when response was detected to the date when progression was observed. The progression-free interval (PFI) was defined as the time from the first day of study treatment to the day of progression or the last day of follow-up. Overall survival was measured from the first day of study treatment to the day of death or last follow-up. A review committee independently reviewed the objective responses and all disease stabilizations.

#### Statistics

All patients (19 patients in arm A and 18 patients in arm B) were analyzed for response. The rate of disease control was calculated as the number of patients who were free of progression for at least 12±1 weeks divided by the number of patients evaluable for response. The rate of objective response was calculated as the number of PRs and CRs, divided by the number of patients evaluable for response. Means and standard deviations, or medians and intervals, were calculated for continuous variables. Absolute frequencies and percentages were calculated for qualitative variables.

Both PFI and survival were calculated according to Kaplan-Meier. Duration of response was calculated in responding patients from the date when response was first detected to the date of progression. If a new treatment was started before progression, duration of response, PFI and survival were censored.

### 2.2. Results

#### Study patients

39 patients were enrolled at eight sites in Spain and Belgium, 19 were randomized to Arm A and 20 were randomized to Arm B. Their median age was 56 (28–75) years. Performance status 0 and 1 were well balanced. There was a male predominace, most patients were Caucasians and all had metastatic disease. 89.7% of patients had previous surgery, 25.6% were pretreated with radiotherapy. Systemic pretreatment included immunotherapy in 74.4% and chemotherapy in 28.2% of patients.

#### Protocol adherence

146 cycles were administered to patients in Arm A (median 4 ; range, 2–49 cycles). The median cumulative dose given to each patient in Arm A was 20.00 mg/m^2^ (range, 8.34–244.76 mg/m^2^) and the  median dose intensity was 2.25 mg/m^2^/week (range, 1.77–2.55 mg/m^2^/week), which corresponded to a median relative dose intensity of 89.89% (range, 70.76–101.95%).

Patients in Arm B received 111 cycles (4 per patient; range, 2–19 cycles). The median cumulative dose given was 26.00 mg/m^2^ (range, 14.00–137.71 mg/m^2^) and the median dose intensity was 3.13 mg/m^2^/week (range, 2.36–3.59 mg/m^2^/week), corresponding to a median relative dose intensity of 89.30% (range, 67.53–102.44%).

Thirteen patients (68.4%) in Arm A and 10 patients (50.0%) in Arm B had one or more cycle delayed. In Arm A, four patients had one cycle delayed, eight had two cycles delayed, and one had three or more cycles delayed. In Arm B, three patients had one cycled delayed, three had two cycles delayed, and four patients had three or more cycles delayed. No patients in Arm A underwent dose reduction. In Arm B, four patients had dose reductions.

#### Duration of treatment and reasons for treatment discontinuation

The median time on treatment was 60 days (28–800 days) for all patients : 63 days (28–800 days) in Arm A, and 57.5 days (28–366 days) in Arm B. Most patients discontinued due to progression: 12 patients (63.1%) in Arm A and 11 patients (55.0%) in B. The second most common cause was toxicity, which affected two (10.5%) and five (25.0%) patients (acute cardiomyopathy after 28 cycles ; muscular toxicity ; muscular weakness and CPK elevation, a combination of musculoskeletal cramps and CPK elevation ; a combination of grade 2 dyspnea, bronchial spams and atrial fibrillation ; three non-plitidepsin-related deaths).

Twenty-five patients with RCC died during the study period: 13 in Arm A and 12 in B. Twenty-two of these deaths were the result of progression. Three patients died unrelated due to other reasons.

### 2.3. Progression free and response rate

Three of 19 evaluable patients in Arm A showed no evidence of progression at week 12 giving an objective rate of disease control of 15.8% (95% CI, 3.4–39.6%). At 12 weeks, 16 patients in arm A (84.2%) and in arm B (88.9%) had disease progression. In Arm B, two of 18 evaluable patients (11,1%) had disease control week 12; (95% CI, 1.4–34.7%). Two patients in Arm A achieved confirmed PR (response rate 10.5%, 95% CI, 1.3–33.1%). They received 49 and 28 treatment cycles and while one of these patient was still on treatment at the cutoff date, the other discontinued the study due to depression unlikely related to the study treatment. Both were metastatic and had failed immunotherapy. The response was achieved after 30 and 24 cycles and the maximum tumor shrinkage in these patients was 41 and 39 %. One patient achieved confirmed stable disease (5.3%) Additionally, seven patients in Arm A had stable disease (SD) that remained unconfirmed at the end of the study.

No patients in Arm B achieved a PR, two had SD (11.1%) as best radiological response that was confirmed in at least two evaluations. They received 16 or 19 cycles and discontinued the study due to PD. Additionally, nine patients achieved unconfirmed SD.

### 2.4. Duration of response, progression-free interval and overall survival

The response in the patients who achieved confirmed PR was 9.51 and 2.5 months (median duration of 6.0 months).

In Arm A, the median PFI was 2.1 months (95% CI, 1.7–2.8 months) and the progression free rate at 3 months was 28.5% (95% CI, 7.5–49.5%), 17.1% (95% CI, 0.0–34.7%) at 6 and 11.4% (95% CI, 0.0–26.2%) at 12 months. In Arm B, the median PFI was 2.1 months (95% CI, 1.7–4.0 months), and the progression free rate at 3 months was 40.8% (95% CI, 17.0–64.6%), 16.3% (95% CI, 0.0–36.3%) at 6, and 8.2% (95% CI, 0.0–23.2%) at 12 months.

In Arm A, the median OS was 7.0 months (95% CI, 4.1–23.2 months) and the survival rates at 6 months was 56.4% (95% CI, 33.6–79.2%), 31.3% (95% CI, 9.1–53.6%) at 12, and 20.9% (95% CI, 0.0–43.2%) at 24 months. In Arm B, median OS was 7.6 months (95% CI, 3.5–19.3 months) and the survival rates at 6 months were 54.8% (95% CI, 29.4–80.2%), 39.1% (95% CI, 13.3–64.9%) at 12, and 23.5% (95% CI, 0.6–46.3%) at 24 months.

### 2.5. Safety

Most AEs were mild or moderate (grade 1 or 2). Grade 3/4 AEs included asthenia (n=3), chest pain (n=1), injection site reaction (n=1), vomiting (n=1) and acute cardiomyopathy (n=1). Treatment-related grade 3/4 AEs were more common in Arm B and included atrial fibrillation (n=1), hiccups (n=1), asthenia (n=1), injection site reaction (n=1), respiratory tract infection (n=1), anorexia (n=1), peripheral motor neuropathy (n=1), bronchospasm (n=1), dyspnea (n=1) and phlebitis (n=1). Severe muscular toxicities included myalgia (n=1) in Arm A, and muscle weakness (n=2), muscle cramps (n=1) and myalgia (n=1) in B.

The treatment-related muscular AEs found in either arm were myalgia and muscle weakness, and their incidence was lower in Arm A (myalgia: 36.8% of patients and 19.2% of cycles; muscle weakness NOS: 26.3% of patients and 7.5% of cycles); Arm B (myalgia: 55.0% of patients and 19.8% of cycles; muscle weakness: 30.0% of patients and 12.6% of cycles). Most cases were mild or moderate. Severe muscular AEs were found in both arms and usually lasted for one cycle only, but  were more common in Arm B (four cases of grade 3/4 muscle weakness, grade 3 muscle cramps or grade 3 myalgia) than in A (one grade 3 myalgia). Two treatment-related cardiac AEs were found with each regimen, but only one patient in Arm A discontinued the study as a result.

CPK elevation was more common in Arm B (45.0% of patients and 12.2% of cycles) than in Arm A (31.6% of patients, 8.4% of cycles). Most cases were mild or moderate, and severe increases were found in one patient and one cycle in each arm. However, whereas three patients in Arm B had to discontinue the study due to events related to CPK elevation alone or concomitant with muscular toxicity, no patients in Arm A did so.

Most treatment-related AEs were mild or moderate, and severe hepatic and muscular toxicity was infrequent and transient. Increasing the plitidepsin dose to 7 mg/m^2^ was associated with a greater incidence of severe treatment-related AEs, non-hematological toxic events (mainly transaminase and CPK elevations) and treatment-related SAEs. Coadministration of L-carnitine did not prevent the occurrence or severity of muscular toxicity and CPK elevation.

### 2.6. Discussion

This phase II study evaluated two regimens of plitidepsin given as a 24-hour i.v. infusion every two weeks to patients with advanced RCC.

Most patients discontinued the treatment due to disease progression, regardless of the regimen administered. Discontinuation due to toxicity was more common with 7 mg/m^2^ plus L-carnitine than with 5 mg/m^2^, and muscular toxicity and/or CPK elevation were the cause of discontinuation of four patients treated with 7 mg/m^2^ plus L-carnitine but none of those treated without L-carnitine. Coadministration of L-carnitine provided no protection against the muscular toxicity associated with a higher dose level of plitidepsin and its use in further studies is not warranted.

The primary objective of this study, which was to determine the effectiveness of plitidepsin, was met. Five patients showed evidence of disease control: two had confirmed PR and one had confirmed SD with 5 mg/m^2^, while two had confirmed SD with 7 mg/m^2^ plus L-carnitine. Based on these results,  the rate of disease control at 12 weeks in all evaluable patients was 15.8% with 5 mg/m^2^ and 11.1% with 7 mg/m^2^ plus L-carnitine. No significant differences were found in the time-related secondary efficacy outcomes; the median PFI was the same and the progression-free rates decreased rapidly over time. The two patients who achieved confirmed PR with 5 mg/m^2^ had PFI values of 13.85+ and 25.86+ months while the patient with confirmed SD with 5 mg/m^2^ had a PFI of 7.34 months and the two patients with confirmed SD with 7 mg/m^2^ plus L-carnitine had PFI values of 9.64 and 12.01 months. The overall results suggest that plitidepsin induces some antitumor activity in selected patients, with better results being achieved with the 5 mg/m^2^ regimen.

The standard of care of advanced RCC has rapidly changed during the conduct of this trial with the development of new therapies such as VEGF inhibitors or the mammalian target of rapamycin inhibitors [19]. The 10.5% response rate achieved in this study with plitidepsin 5 mg/m^2^ is similar to the 10–15% found with traditional standards such as IFN-alpha or high-dose IL-2 [20,21] and to the 11% reported for sorafenib [22], although lower than the response rate found with sunitinib [26] or bevacizumab combined with IFN [24].

Other potential new therapies have been chosen for further development based on survival improvement in spite of low response rates. For instance, phase II trials in patients with metastatic refractory RCC showed that temsirolimus induced a response rate of only 7% but a median survival of 15 months [25]. Numerous studies are currently assessing different combinations of active agents in this setting.

The antitumor activity of plitidepsin, similar to that of some approved single agents together with its unique effects on VEGF secretion which have not been described with other agents used for treatment of RCC open the way to future studies evaluating the potential of combining this marine compound with therapies currently available for the treatment of RCC.

## Figures and Tables

**Figure 1:**
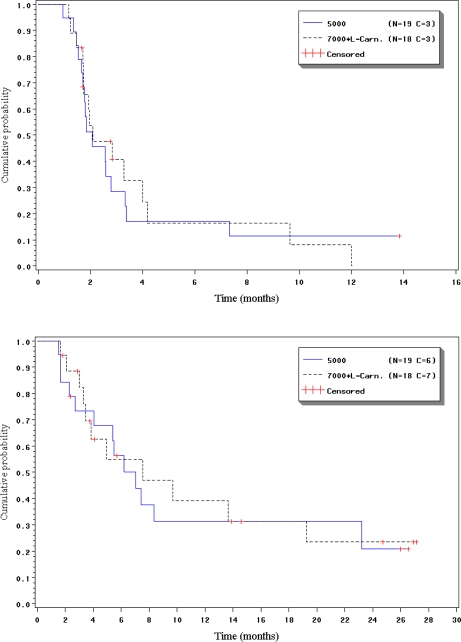
Progression-free and overall survival (Kaplan-Meier).

**Table 1:** Patient and tumor characteristics.

	Arm A		Arm B		Total	
	
	n	%	n	%		%

No. of patients	19	100.0	20	100.0	39	100.0

**Gender**						
Male	13	68.4	16	80.0	29	74.4
Female	6	31.6	4	20.0	10	25.6

**Race**						
Caucasian	19	100.0	18	90.0	37	94.9
Hispanic	.	.	2	10.0	2	5.1

**Age (years)**						
Median	56.0	.	56.0	.	56.0	.
Range	28.0–75.0	.	42.0–74.0	.	28.0–75.0	.

**ECOG PS**						
0	10	52.6	10	50.0	20	51.3
1	9	47.4	10	50.0	19	48.7

**Histology of primary tumor**						
Chromophobic	1	5.3	.	.	1	2.6
Clear cell carcinoma	16	84.1	19	95.0	35	89.6
Epidermoid *and* clear cell carcinoma	.	.	1	5.0	1	2.6
Mixed clear cell carcinoma	1	5.3	.	.	1	2.6
Papillary carcinoma	1	5.3	.		1	2.6

**No. of metastatic sites**	2	.	2	.	2	.
Median (range)	(1–5)	.	(1–5)	.	(1–5)	.

**Time from diagnosis to plitidepsin (months)**		.				
Median	13.8		19.1	.	13.8	.
Range	1.0–109.5	.	1.0–109.9	.	1.0–109.9	.

**Surgery**						
Yes	16	84.2	19	95.0	35	89.7
No	3	15.8	1	5.0	4	10.3

**Radiotherapy**						
Yes	4	21.1	6	30.0	10	25.6
No	15	78.9	14	70.0	29	74.4

**Immunotherapy**						
Yes	14	73.7	15	75.0	29	74.4
No	5	26.3	5	25.0	10	25.6

**Prior immunotherapy agents**	19	100.0	18	100.0	37	100.0
n	7	36.8	11	61.1	18	48.6
Interferon	7	36.9	4	22.2	11	29.8
Interleukin-2	5	26.3	3	16.7	8	21.6

**Chemotherapy**						
Yes	5	26.3	6	30.0	11	28.2
No	14	73.7	14	70.0	28	71.8

**Table 2:** Treatment-related adverse events in Arm A and B (worst grade per patient).

ARM A	NCI-CTC grade					
	1		2		3	
	n	%	n	%	n	%
**Constipation**	2	10.5	.	.	.	.
**Diarrhea**	5	26.3	.	.	.	.
**Nausea**	10	52.6	4	21.1	.	.
**Vomiting**	9	47.4	2	10.5	1	5.3
**Asthenia**	4	21.1	6	31.6	3	15.8
**Edema**	3	15.8	.	.	.	.
**Injection site reaction**	3	15.8	2	10.5	1	5.3
**Weight decreased**	2	10.5	.	.	.	.
**Anorexia**	7	36.8	2	10.5	.	.
**Muscle cramps**	2	10.5	1	5.3	.	.
**Muscle weakness**	2	10.5	3	15.8	.	.
**Myalgia**	3	15.8	3	15.8	1	5.3
**Dizziness**	2	10.5	.	.	.	.
**Folliculitis**	2	10.5	.	.	.	.
**Rash**	1	5.3	.	.	.	.
**ARM B**	**NCI-CTC grade**					
	**1**		**2**		**3**	
	n	%	n	%	n	%
**Constipation**	2	10.0	.	.	.	.
**Diarrhea**	4	20.0	3	15.0	.	.
**Nausea**	7	35.0	9	45.0	.	.
**Stomatitis**	2	10.0	.	.	.	.
**Vomiting**	10	50.0	6	30.0	.	.
**Asthenia**	2	10.0	10	50.0	1	5.0
**Injection site reaction**	3	15.0	2	10.0	1	5.0
**Anorexia**	6	30.0	3	15.0	1	5.0
**Myalgia**	5	25.0	5	25.0	1	5.0

**Table 3:** Hematological toxicity in Arm A and B (worst grade per patient).

	NCI-CTC grade					
	1		2		3	
	n	%	n	%	n	%
**Arm A**						
**Hemoglobin**	7	36.8	11	57.9	1	5.3
**Lymphocytes**	2	10.5	3	15.8	3	15.8
**Neutrophils**	1	5.3	.	.	.	.
**Platelets**	.	.	1	5.3	1	5.3
**WBC**	.	.	1	5.3	.	.
**Arm B**						
**Hemoglobin**	12	60.0	6	30.0	1	5.0
**Lymphocytes**	.	.	3	15.8	2	10.5
**Neutrophils**	1	5.3	.	.	.	.
**Platelets**	3	15.8	.	.	1	5.3
**WBC**	2	10.5	.	.	.	.

**Table 4:** Biochemical toxicity in Arm A and B (worst grade per patient).

	NCI-CTC grade							
	1		2		3		4	
	n	%	n	%	n	%	n	%
**Arm A**								
**ALT**	5	26.3	3	15.8	4	21.1	.	.
**AP**	6	31.6	3	15.8	.	.	.	.
**AST**	9	47.4	2	10.5	2	10.5	.	.
**CPK**	3	15.8	2	10.5	.	.	1	5.3
**Creatinine**	10	52.6	2	10.5	.	.	.	.
**GGT**	4	21.1	4	21.1	7	36.8	.	.
**LDH**	10	55.6	2	11.1	.	.	1	5.6
**Total bilirubin**	3	15.8	.	.	.	.	.	.
**Hypoalbuminemia**	9	52.9	4	23.5	.	.	.	.
**Hypercalcemia**	7	36.8	.	.	1	5.3	.	.
**Hypocalcemia**	2	10.5	6	31.6	.	.	.	.
**Hyperglycemia**	11	57.9	4	21.1	2	10.5	.	.
**Hypoglycemia**	1	5.3	1	5.3	.	.	.	.
**Hyperkalemia**	2	10.5	3	15.8	1	5.3	.	.
**Hypokalemia**	5	26.3	.	.	2	10.5	.	.
**Hypernatremia**	2	10.5	.	.	.	.	.	.
**Hyponatremia**	6	31.6	.	.	1	5.3	1	5.3
**Arm B**								
**ALT**	6	30.0	3	15.0	6	30.0	.	.
**AP**	12	60.0	1	5.0	.	.	.	.
**AST**	5	25.0	6	30.0	1	5.0	.	.
**CPK**	5	25.0	3	15.0	1	5.0	.	.
**Creatinine**	8	40.0	4	20.0	2	10.0	.	.
**GGT**	5	25.0	6	30.0	4	20.0	2	10.0
**LDH**	10	50.0	2	10.0	.	.	.	.
**Total bilirubin**	1	5.0	1	5.0	1	5.0	.	.
**Hypoalbuminemia**	6	31.6	4	21.1	.	.	.	.
**Hypercalcemia**	7	35.0	.	.	1	5.0	.	.
**Hypocalcemia**	6	30.0	2	10.0	1	5.0	1	5.0
**Hyperglycemia**	8	40.0	5	25.0	3	15.0	.	.
**Hypoglycemia**	1	5.0	2	10.0	.	.	.	.
**Hyperkalemia**	4	20.0	.	.	3	15.0	1	5.0
**Hypokalemia**	5	25.0	.	.	1	5.0	.	.
**Hypernatremia**	2	10.0	.	.	.	.	.	.
**Hyponatremia**	6	30.0	.	.	1	5.0	.	.

AP, alkaline phosphatase; ALT, alanine aminotransferase; AST, aspartate aminotransferase; CPK, creatine phosphokinase; GGT, gamma-glutamyltransferase; LDH, lactate dehydrogenase.

## References

[1] (1996). Cancer Facts & Figures - 1996.

[2] Linehan WM, Shipley W, Parkinson D, De Vita VTJ, Hellman S, Rosenberg SA (1997). Cancer of the kidney and ureter. Cancer: Principles and Practice of Oncology.

[3] Maher ER, Iselius L, Yates JR, Littler M, Benjamin C, Harris R, Sampson J, Williams A, Ferguson-Smith MA, Morton N (1991). Von Hippel-Lindau disease: a genetic study. J. Med. Genet.

[4] Pisters LL, El-Naggar AK, Luo W, Malpica A, Lin SH (1997). C-met proto-oncogene expression in benign and malignant human renal tissues. J. Urol.

[5] Gnarra JR, Zhou S, Merrill MJ, Wagner JR, Krumm A, Papavassiliou E, Oldfield EH, Klausner RD, Linehan WM (1996). Post-transcriptional regulation of vascular endothelial growth factor mRNA by the product of the VHL tumor suppressor gene. Proc. Natl. Acad. Sci. USA.

[6] Sene AP, Hunt L, McMahon RF, Carroll RN (1992). Renal carcinoma in patients undergoing nephrectomy: analysis of survival and prognostic factors. Br. J. Urol.

[7] Motzer RJ, Bander NH, Nanus DM (1996). Renal-cell carcinoma. N. Engl. J. Med.

[8] Negrier S, Escudier B, Lasset C, Douillard JY, Savary J, Chevreau C, Ravaud A, Mercatello A, Peny J, Mousseau M, Philip T, Tursz T (1998). Recombinant human interleukin-2, recombinant human interferon alfa-2a, or both in metastatic renal-cell carcinoma. Groupe Francais d'Immunotherapie. N. Engl. J. Med.

[9] Bukowski RM (1997). Natural history and therapy of metastatic RCC: the role of interleukin-2. Cancer.

[10] Rosenberg SA, Lotze MT, Muul LM, Chang AE, Avis FP, Leitman S, Linehan WM, Robertson CN, Lee RE, Rubin JT (1987). A progress report on the treatment of 157 patients with advanced cancer using lymphokine-activated killer cells and interleukin-2 or high-dose interleukin-2 alone. N. Engl. J. Med.

[11] Biscadi M, Caporale R, Balestri F, Gavazzi S, Jimeno J, Grossi A (2005). VEGF inhibition and cytotoxic effect of aplidin in leukemia cell lines and cells from acute myeloid leukemia. Ann. Oncol.

[12] Faircloth G, Rinehart K, Nunez DC (1996). Dehydrodidemnin B a new marine derived antitumor agent with activity against experimental tumor models. Ann. Oncol.

[13] Casciari JJ, Hollingshead MG, Alley MC, Mayo JG, Malspeis L, Miyauchi S, Grever MR, Weinstein JN (1994). Growth and chemotherapeutic response of cells in a hollow-fiber *in vitro* solid tumor model. J. Natl. Cancer Inst.

[14] Anthoney A, Paz-Ares L, Twelves C (2000). Phase I and pharmacokinetic (PK) study of Aplidin (APL) using a 24-hour, weekly schedule. Proc Am Soc Clin Oncol.

[15] Izquierdo MA, Bowman A, Garcia M, Jodrell D, Martinez M, Pardo B, Gómez J, López-Martin JA, Jimeno J, Germá JR, Smyth JF (2008). Phase I clinical and pharmacokinetic study of plitidepsin as a 1-hour weekly intravenous infusion in patients with advanced solid tumors. Clin. Cancer Res.

[16] Faivre S, Chièze S, Delbaldo C, Ady-Vago N, Guzman C, Lopez-Lazaro L, Lozahic S, Jimeno J, Pico F, Armand JP, Martin JA, Raymond E (2005). Phase I and pharmacokinetic study of aplidine aplidine, a new marine cyclodepsipeptide, in patients with advanced malignancies. J. Clin. Oncol.

[17] Maroun JA, Belanger K, Seymour L, Ady-Vago N, Guzman C, Lopez-Lazaro L, Lozahic S, Jimeno J, Pico F, Armand JP, Martin JA, Raymond E (2006). Phase I study of Aplidine in a dailyx5 one hour infusion every 3 weeks in patients with solid tumors refractory to standard therapy.A National Cancer Institute of Canada Clinical Tirals Group Study : NCIC CTG IND 115. Ann. Oncol.

[18] Cumming WJ, Hardy M, Hudgson P, Walls J (1976). Carnitine-palmityl-transferase deficiency. J. Neurol. Sci.

[19] Brugarolas J (2007). Renal-cell carcinoma–molecular pathways and therapies. N. Engl. J. Med.

[20] Speca J, Yenser S, Creel P, George D (2006). Improving outcomes with novel therapies for patients with newly diagnosed RCC. Clin. Genitourin Cancer.

[21] Rock EP, Goodman V, Jiang JX, Mahjoob K, Verbois SL, Mors D, Dagher R, Justice R, Pazdur R (2007). Food and Drug Administration drug approval summary: Sunitinib malate for the treatment of gastrointestinal stromal tumor and advanced RCC. Oncologist.

[22] Escudier B, Pluzanska A, Koralewski P, Ravaud A, Bracarda S, Szczylik C, Chevreau C, Filipek M, Melichar B, Bajetta E, Gorbunova V, Bay JO, Bodrogi I, Jagiello-Gruszfeld A, Moore N (2007). AVOREN Trial investigators. Bevacizumab plus interferon alfa-2a for treatment of metastatic renal cell carcinoma : a randomised, double-blind phase III trial. Lancet.

[23] Atkins MB, Regan M, McDermott DF (2004). Update on the role of interleukin 2 and other cytokines in the treatment of patients with stage IV renal carcinoma. Clin. Cancer Res.

[24] McDermott DF, Atkins MB (2004). Application of IL-2 and other cytokines in RCC. Expert Opin. Biol. Ther.

[23] Atkins MB, Hidalgo M, Stadler WM, Loga TF, Dutcher JP, Hudes GR, Park Y, Liou SH, Marshall B, Boni JP, Dukart G, Sherman ML (2004). Randomized phase II study of multiple dose levels of CCI-779, a novel mammalian target of rapamycin kinase inhibitor, in patients with advanced refractory RCC. J. Clin. Oncol.

